# The interaction between stimulus factors and cognitive factors during multisensory integration of audiovisual speech

**DOI:** 10.3389/fpsyg.2014.00352

**Published:** 2014-05-01

**Authors:** Ryan A. Stevenson, Mark T. Wallace, Nicholas Altieri

**Affiliations:** ^1^Psychology Department, University of TorontoToronto, ON, Canada; ^2^Vanderbilt Brain Institute, Vanderbilt University Medical CenterNashville, TN, USA; ^3^Department of Hearing and Speech Sciences, Vanderbilt University Medical CenterNashville, TN, USA; ^4^Vanderbilt Kennedy Center, Vanderbilt University Medical CenterNashville, TN, USA; ^5^Department of Psychology, Vanderbilt UniversityNashville, TN, USA; ^6^Department of Psychiatry, Vanderbilt University Medical CenterNashville, TN, USA; ^7^Department of Communication Sciences and Disorders, Idaho State UniversityPocatello, ID, USA

**Keywords:** multisensory processing, audiovisual integration, speech perception, temporal processing, sensory processing, crossmodal, perceptual binding, speech integration

The amount of research focused on multisensory speech perception has expanded considerably in recent years. Much of this research has focused on which factors influence whether or not an auditory and a visual speech input are “integrated” (i.e., perceptually bound); a special case of how our perceptual systems solve the “binding problem” (Treisman, 1996). The factors that have been identified as influencing multisensory integration can be roughly divided into two groups. First are the low-level stimulus factors that include the physical characteristics of the sensory signals. The most commonly studied of these include the spatial (e.g., Macaluso et al., 2004; Wallace et al., 2004) and temporal (e.g., Miller and D'Esposito, 2005; Stevenson et al., 2011) relationship of the two inputs, and their relative effectiveness (e.g., James et al., 2012; Kim et al., 2012) in driving a neural, perceptual, or behavioral response. The second group of factors can be considered more higher-order or cognitive, and include factors such as the semantic congruence of the auditory and visual signals (Laurienti et al., 2004) or whether or not the gender of the speaker's voice is matched to the face (Lachs and Pisoni, 2004).

While these two categories can be considered conceptually distinct, they are related because of their mutual dependence upon the natural statistics of signals in the environment. When auditory and visual speech signals are closely proximate in time (low-level), they are more likely to have originated from the same speaker, and thus should be integrated (Dixon and Spitz, 1980; Stevenson et al., 2012b). Likewise, if an auditory and a visual speech signal are semantically congruent (high-level), they are more likely to have originated from the same speaker and thus should be integrated (Calvert et al., 2000). Given that these low- and high-level factors are each reflective of the natural statistics of the environmental signals, they will generally co-vary. Taking speech as an example, in a natural setting, the temporally-coincident auditory and visual components of a syllable or word are also semantically congruent (Spence, 2007).

To date, most research has investigated these low- and high-level factors independently. These studies have been highly informative, providing descriptions as to how each of these factors contributes to the process of multisensory integration. What has not received a great deal of focus is the interplay between these factors. A handful of experiments have investigated how low-level factors interact with one another and influence multisensory integration (Macaluso et al., 2004; Royal et al., 2009; Stevenson et al., 2012a), but few have attempted to bridge between low-level stimulus-characteristics and high-level cognitive factors (Vatakis and Spence, 2007). A recent article by Ten Oever et al. (2013), *Audio-visual onset differences are used to determine syllable identity for ambiguous audio-visual stimulus pairs* addresses this gap in our understanding by investigating the interaction between stimulus timing and semantic congruency modulated by changes in place of articulation or voicing.

In this study, participants were presented with single-syllable stimuli, with auditory, visual, and audiovisual syllables systematically manipulated according to place of articulation and voicing. In addition, the temporal alignment of the audiovisual presentations was also parametrically varied. Hence, semantic content was varied through changes both in the auditory (voicing) and visual (place of articulation) signals, while at the same time, the relative timing of the auditory and visual stimuli were systematically varied. While the results specific to these factors are interesting on their own, most germane to this commentary is how these two factors interacted. The authors measured the window of time within which the visual cue influenced the syllable that was heard. This probabilistic construct, referred to as the “time window of integration” or the “temporal binding window,” has been shown to vary greatly according the type of stimulus being integrated (Vatakis and Spence, 2006; Stevenson and Wallace, 2013). In the Ten Oever et al. study, semantically congruent stimuli were found to be associated with a wider temporal binding window than semantically incongruent stimuli. That is, stimulus components that are semantically matched have higher rates of integration at more temporally disparate offsets.

The result is surprising in that it runs counter to predictions generated by hierarchical serial models. In such models, lower-level properties such as stimulus timing are processed initially, and are then followed by the processing of the linguistic (i.e., semantic) content in the auditory and visual signals. However, the current results, by illustrating an interaction between timing and congruency, suggest that hierarchical models are insufficient to explain the data. Rather, we posit that these results are better interpreted within a “parallel accumulation of evidence” framework (Figure 1). In this model, the temporal relationship of two sensory inputs provides important information about the likelihood that those two inputs originated from the same speaker and should be integrated. In addition, the semantic congruence of these inputs also provides information as to whether or not the two sensory inputs should be bound. Importantly, these two types of evidence are pooled into a single decision criterion. Thus, within such a framework, when stimuli are semantically congruent, a decreased amount of temporal alignment is needed in order to cross a decision bound that would result in these two inputs being integrated, manifesting in a broader temporal binding window for semantically congruent speech stimulus pairs.

**Figure 1:**
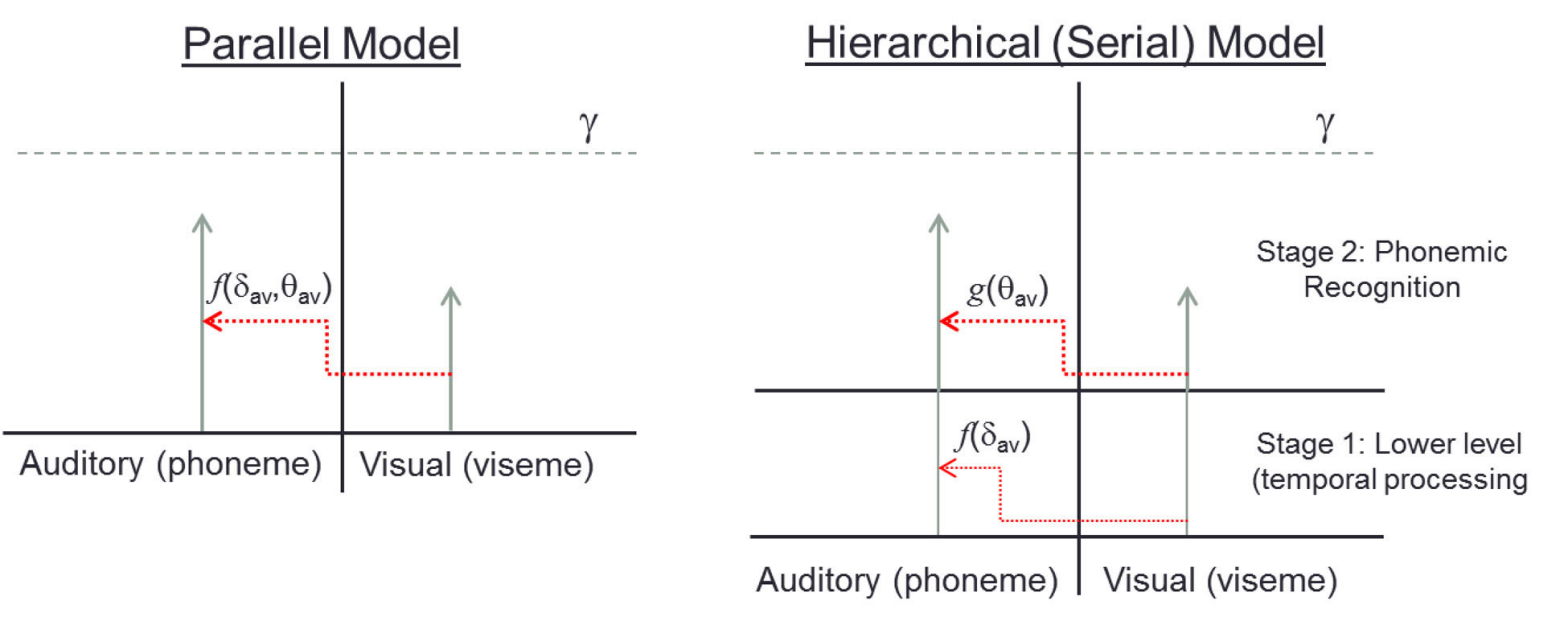
**The left panel shows a “parallel accumulator” model with auditory and visual evidence racing toward threshold (γ).** The amount of visual influence on the auditory signal is a function, *f*, of parameters δ, and θ which represent temporal coincidence detection and phonetic congruence respectively, both of which contribute evidence to a single accumulator. The serial model on the right shows two separate stages where integration is affected first by temporal, then by semantic processing. Hence, in stage 1, visual information influences auditory processing only as a function *f* of temporal coincidence. In stage 2, visual information influences auditory processing solely as a function,*g*, of phonemic compatibility.

Through this interaction between stimulus timing and semantic congruence, Ten Oever and colleagues provided compelling evidence that low-level stimulus and high-level cognitive factors are not processed in a completely serial manner, but rather interact with one another in the formation of a perceptual decision. These results have significant implications in informing our view as to the neurobiological substrates involved in real-world multisensory perceptual processes. Most importantly, the work suggests that significant feedforward and feedback circuits are engaged in the processing of naturalistic multisensory stimuli, and that these circuits work in a parallel and cooperative fashion in evaluating the statistical relations of the stimuli to one another on both their low-level (i.e., stimulus feature) and high-level (i.e., learned semantic) correspondences.

## Conflict of interest statement

The authors declare that the research was conducted in the absence of any commercial or financial relationships that could be construed as a potential conflict of interest.
